# Novel compound heterozygous mutations in the *PARK2* gene identified in a Chinese pedigree with early‐onset Parkinson's disease

**DOI:** 10.1002/brb3.901

**Published:** 2017-12-19

**Authors:** Yingying Shi, Hideshi Kawakami, Weizhou Zang, Gang Li, Jiewen Zhang, Changshui Xu

**Affiliations:** ^1^ Department of Neurology Henan Provincial People's Hospital Zhengzhou China; ^2^ Department of Epidemiology Research Institute for Radiation Biology and Medicine Hiroshima University Hiroshima Japan

**Keywords:** gross deletions, intronic splice site mutations, Parkinson's disease

## Abstract

**Objects:**

To capture point mutations and short insertions/deletions in 49 previously reported genes associated with Parkinson's disease (PD) in a Chinese pedigree with early‐onset Parkinson's disease (EOPD)‐affected individuals.

**Methods:**

Clinical examinations and genomic analysis were performed on 21 subjects belonging to three generations of a Chinese family. Target region capture and high‐throughput sequencing were used for screening 49 genes, which were previously reported to be associated with PD. The direct Sanger sequencing method in all subjects further verified the abnormal DNA fragments in the *PARK2* gene.

**Results:**

Four family members, including a mother (I‐1) and her three children (II‐2, II‐3, and II‐7), were diagnosed with PD by clinical manifestations and/or PET/CT imaging analyses. Novel compound heterozygous mutations, consisting of a fragment deletion in exon 1 to 2 (EX 1‐2 del) and a splicing point mutation c.619‐1 (G > C) in the 6th intron of the *PARK2* gene, were identified in II‐2, II‐3, and II‐7. Individual EX 1‐2 del or c.619‐1 (G > C) mutations were detected in I‐1 and the third generation (III‐2, 3, 5, 10, and 11).Other mutations were not detected in the 49 known PD‐associated genes.

**Conclusion:**

Novel compound heterozygous mutations were identified in a Chinese pedigree and might represent a cause of familial EOPD with autosomal dominant inheritance.

## 1 INTRODUCTION

First described in 1817, Parkinson's disease (PD) is one of the most frequent progressive neurodegenerative disorders, with a prevalence of approximately 1.7% in people over 65 years of age (de Rijk et al., 1997). It is caused by the progressive loss of dopaminergic neurons and characterized by motor symptoms such as rigidity, bradykinesia, resting tremor, and postural instability. Although the etiology of PD has not been fully elucidated, epidemiological studies have suggested that both genetic and environmental factors play important roles in the pathogenesis of PD. Recent studies have identified several genes such as *PARK2, PARK7, LRRK2, SCNA, UCHL1,* and *PINK1* whose mutations could result in rare familial forms of PD. Among them, loss‐of‐function mutations in the gene designated parkin in the *PARK2* locus accounted for about 50% of familial autosomal recessive EOPDs (Kitada et al., 1998; Leroy, Anastasopoulos, Konitsiotis, Lavedan, & Polymeropoulos, 1998; Lucking et al., 2000; Periquet et al., 2003).

The parkin gene (*PARK2*) is one of the largest genes in the genome, which contains 12 exons with a superexpanded intronic structure spanning 1.3 Mb, and encodes 465 amino acids, with domains characteristic of ubiquitin‐conjugating enzymes (E2s) Ubch7 or Ubch8 (Shimura et al., 2000). It is likely that the variations in the *PARK2* gene could lead to conformational and functional changes in parkin. However, in the past few years, direct sequencing analysis of 51 genomic DNA samples of the *PARK2* gene did not reveal any direct evidence to reach unequivocal conclusions (Nuytemans, Theuns, Cruts, & Van Broeckhoven, 2010). *PARK2* shares a common promoter with *parkin coregulated gene* (*PACRG*), which contains six exons and encodes 257 amino acids (West, Lockhart, O'Farell, & Farrer, 2003). It lies upstream of *PARK2* in a head‐to‐head orientation and patients with *PACRG* mutations present with similar severity disorders as other EOPD patients with *PARK2* mutations (Lesage et al., 2007; West et al., 2003).

Here, we present novel compound heterozygous mutations in the *PARK2* gene, consisting of a gross deletion (EX 1‐2 del; deletion in the exon 1 to 2) along with a splicing mutation c.619‐1 (G > C) in a Chinese pedigree with EOPD‐affected members detected using next‐generation sequencing.

## 2 MATERIALS AND METHODS

### 2.1 Subjects

A third‐generation, 20 subjects Chinese pedigree was examined in this study. The grandmother (I‐1) and three of her children (II‐2, 3 and 4) were diagnosed as PD patients according to clinical manifestations and physical examinations in the Henan Provincial People's Hospital; the other 16 family members had no clinical manifestations of PD. The study was approved by the ethical committee of the Henan Provincial People's Hospital, and written informed consent was obtained from all patients. Our study was performed in accordance with the Declaration of Helsinki regarding the ethical principles for medical research involving human subjects.

### 2.2 Genomic and molecular analyses

A total of 5 ml venous blood from each subject was drawn into sterile EDTA‐anticoagulant tubes, and the whole‐genome library established from the DNA extracted using a DNA Blood Mini Kit (Qiagen, Hilden, Germany). For proband II‐2, target region capture and high‐throughput sequencing were examined for 49 previously reported genes associated with PD.

Analyses of point mutations and deletions were performed using the multiplex ligation‐dependent probe amplification (MLPA) method for the detection of rearrangements of *PARK2* exons. In brief, the specific biotinylated probes were mixed with the genomic library followed by hybridization of target region‐related gene fragments and the biotinylated probes. The target DNA fragments were adsorbed onto biotin and streptavidin magnetic beads and separated. The eluent was further processed with a next‐generation sequencing (NGS) machine (Illumina HiSeq 2500, USA).

Direct sequencing identified point mutations in the parkin gene with Sanger sequencing. We designed the target site primer based on the location of the *PARK2* gene variant. The polymerase chain reaction (PCR) primers were as follows: forward 5′‐GTTCCTGGGAAAGGTTTGATGC‐3′ and reverse 5′‐ GAGTTCACTGAGGAAGGCTCG‐3′. After PCR amplification, the PCR products were purified and sequenced using an ABI3100 genetic analyzer (Applied Biosystems, Foster City, CA, USA). Sequencing reads and lists were compared using Chromas software and NCBI blast.

#### 2.2.1 Brain imaging

Positron emission tomography (PET) followed by computed tomography (CT) was conducted for the proband (II‐2) and one of her brothers (II‐7). Brain PET imaging was performed 30 min after injection of 11C 2 β‐carbomethoxy‐3 β‐(4‐fluorophenyl) tropane (11C‐CFT) and 18F‐2‐fluoro‐2‐deoxy‐D‐glucose (18F‐FDG). Radioactive uptake of 11C‐CFT and 18F‐FDG was calculated using region of interest (ROI) techniques based on CT imaging and evaluated with the Philips Gemini GXL 16 PET/CT scanner. 18F‐FDG brain metabolic imaging and 11C‐CFT brain dopamine transporter PET imaging were performed 2 days after injection to exclude interference between the two imaging agents.

## 3 RESULTS

### 3.1 Chinese family pedigree

A third‐generation, 20 subjects in total, Chinese family was investigated in the present study. The 59‐year‐old woman (proband, II‐2) first manifested resting tremor from the right upper extremity when she was 24 years old in 1987 and developed postural instability with a leaning forward gait. Over the following 3 years, she developed slow progressive myotonia and was diagnosed as PD in 1990 by neurologists according to criteria consistent with the UK Parkinson's Disease Society Brain Bank. She was given a single dose of levodopa–benserazide (62.5 mg tid), which was gradually increased to 125 mg q8 h combined with trihexyphenidyl (2 mg bid) and piribedil (50 mg tid) due to increased age and progression of the illness. In contrast, her mother (I‐1) manifested onset of dystonia when she was 58 years old in 1986 and was also diagnosed with PD in 1990, when she developed postural instability. No medication was prescribed to the mother (I‐1), and she was able to take care of herself until the last follow‐up in 2016. Early onset of resting tremor occurred in II‐3 and II‐7 when they were in the mid‐twenties. Postural instability also subsequently developed and the diagnosis of PD was made several years later when muscular rigidity appeared. Both II‐3 and II‐7 showed consistent and good responses to levodopa. The main symptoms of the PD patients are shown in Table 1.

**Table 1:** Main symptoms of the four PD patients

	I‐1	II‐2	II‐3	II‐7
Sensitivity to dopamine	−	+	+	+
Resting tremor	−	+	+	+
Myotonia	+	+	+	+
Bradykinesia	−	+	+	+
Posture balance disturbance	+	+	+	+

### 3.2 Brain imaging

**Figure 1:**
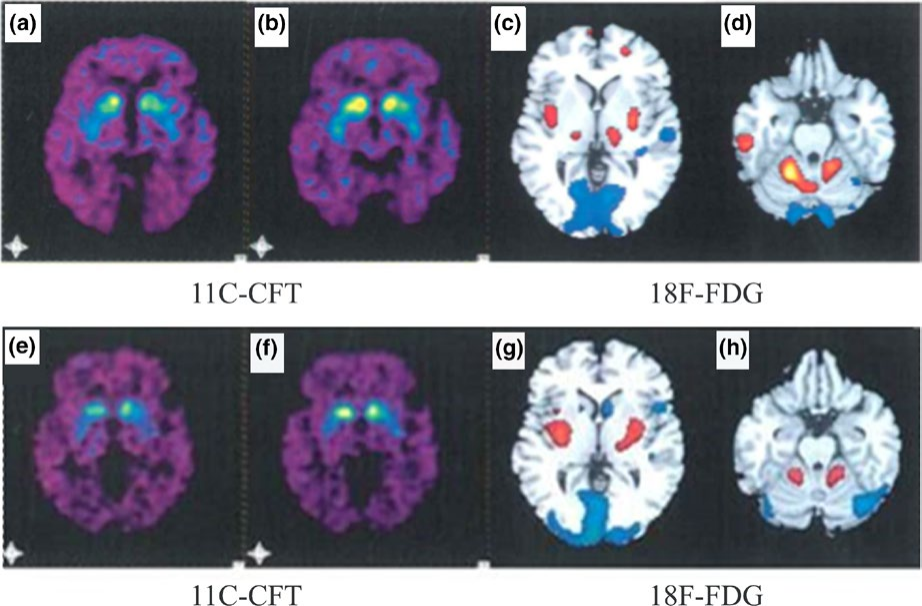
11C‐CTF and 18F‐FDG PET/CT brain images of II‐2 (a‐d) and II‐7 (e‐g)

In addition, II‐2 and II‐7 patients have been examined by PET, and the brain 18F‐FDG and 11C‐CFT imaging revealed PD‐related pattern. The dopamine transporter (DAT) imaging of II‐2 showed that 11C‐CFT uptake was reduced particularly in the caudal putamen (Figure 1a,b). In addition, unequal radioactive distribution was detected by PET/CT imaging after intravenous injection of 18F‐FDG under fasting. 18F‐FDG metabolism increased in the putamen and cerebellum, but no obvious increase of 18F‐FDG metabolism was found in the parietal, temporal, and occipital lobes (Figure 1c,d). Similar distributions of radioactive tracers were found in one of the proband brothers (II‐7) (Figure 1e,f,g,h).

### 3.3 Mutation analysis

**Figure 2:**
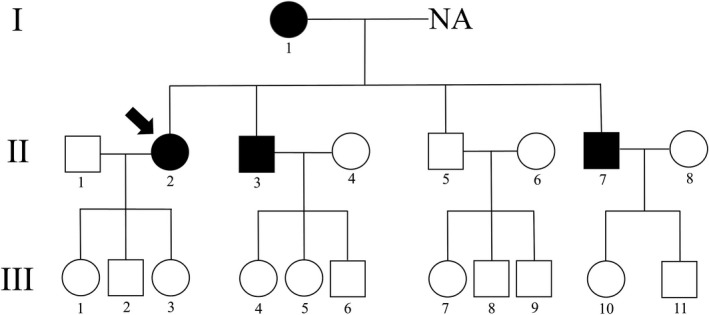
Pedigree of PD. The arrow indicates the first proband for DNA screening. Circle: female; black circle: female patients; square: male; black square: male patients; NA: not analyzed, since the husband deceased before the study

As shown in Figure S1, there were two regions on chromosome 6 corresponding to the *PARK2* gene location in II‐2, whose fluorescence signal intensity was <0.75 (0.75‐1.25 was regarded as a normal range), suggesting a fragment deletion on exons 1 to 2 (EX 1‐2 del) of the *PARK2* gene. In addition, a heterozygous splicing mutation on the ‐1 nt of intron 6 c.619‐1 (G > C) in the *PARK2* gene was detected by direct Sanger sequencing, which has not been reported in the Human Genetics Mutation Database (HGMD, http://www.hgmd.org. 2016). Same mutations, consisting of EX 1‐2 del and c.619‐1 (G > C) in the 6th intron of the *PARK2* gene, were also detected in two of the proband's brothers (II‐3 and II‐7). In comparison, either only the EX 1‐2 del or only the c.619‐1 (G > C) mutation in intron 6 of the *PARK2* gene was detected in III‐2, 3, 5, 11, and I‐1, III‐10, respectively (Table 2, Figure 2).

**Table 2:** Mutation analyses of the Chinese pedigree

	Gender	Exon 1 deletion	Exon 2 deletion	Heterozygous mutation c.619‐1 (G > C)
I‐1	Female	No	No	Yes
I‐2	Male (died)			
II‐1	Male	No	No	No
II‐2	Female	Yes	Yes	Yes
II‐3	Male	Yes	Yes	Yes
II‐4	Female	No	No	No
II‐5	Male	No	No	No
II‐6	Female	No	No	No
II‐7	Male	Yes	Yes	Yes
II‐8	Female	No	No	No
III‐1	Female	No	No	No
III‐2	Male	Yes	Yes	No
III‐3	Female	Yes	Yes	No
III‐4	Female	No	No	No
III‐5	Female	Yes	Yes	No
III‐6	Male	No	No	No
III‐7	Female	No	No	No
III‐8	Male	No	No	No
III‐9	Male	No	No	No
III‐10	Female	No	No	Yes
III‐11	Male	Yes	Yes	No

## 4 DISCUSSION

This study has shown that mutations in the *PARK2* gene are the cause of PD in the Chinese family investigated, which is the most common cause of autosomal recessive PD (Lucking et al., 2000; Periquet et al., 2003).

As the descriptions of Japanese families with heterozygous exon deletions have been reported (Kitada et al., 1998), many mutations have been identified through the entire open reading frame (ORF) of the parkin gene (Hedrich et al., 2004), which has been shown to be present in 50% of familial EOPD patients and responsible for 10‐20% of cases worldwide (Corti, Lesage, & Brice, 2011). Among these wide varieties of mutations identified, more than half were small (point) mutations, and the others were large fragment deletions or duplications (Hedrich et al., 2004; Oliveira et al., 2003). The latter could be much more pathogenic due to derangement of protein structures. For example, in a cohort survey, *PARK2* exonic rearrangements were present in more than 10.8% of Iranian patients (Guo et al., 2010), and similar frequencies have also been reported in Italian, Korean, Belgian, and Chinese populations (Guo et al., 2015; Kim et al., 2012; Nuytemans et al., 2009; Scarciolla et al., 2007). In the current study, we screened point mutations and gross deletions in 49 known PD‐associated genes in the proband II‐2 and identified novel heterozygous *PARK2* mutations, which appeared in three EOPD patients of the Chinese family.

Mutations in *PARK2* play important roles in the pathogenesis of autosomal recessive juvenile parkinsonism (AR‐JP). About half of PD patients with *PARK2* mutations were reported to have either homozygous or compound heterozygous exon rearrangements (Hedrich et al., 2004; Lincoln et al., 2003; Oliveira et al., 2003; Spatola & Wider, 2014). Studies have suggested that one heterozygous deletion or duplication was insufficient and a second mutation was required (Kay et al., 2010; Lincoln et al., 2003). However, other studies have shown that heterozygous mutations in this gene might be a risk factor for the development of PD (Huttenlocher et al., 2015).The novel compound heterozygous mutations in *PARK2* reported in this study included a gross deletion of exons 1 to 2 (EX 1‐2 del) and a splicing mutation (c.619‐, G > C), without any other known PD‐related mutation.

We analyzed family members with PD in the current study and found that single heterozygous mutations were also detected both in PD patients and individuals with no symptoms. The compound heterozygous mutations consisting of deletions spanning the exons 1 to 2 (EX 1‐2 del) and the splicing mutation c.619‐1 (G > C) in the *PARK2* gene, which was firstly identified in the proband (II‐2) who had an EOPD at 29 years of age. The same compound heterozygous mutation carriers (II‐3 and II‐7) also had classical Parkinson symptoms such as resting tremor, hypokinetic rigidity, and bradykinesia with an onset <30 years of age. The grandmother (I‐1) with the single splicing mutation c.619‐1 (G > C) manifested only rest tremors at the age of 58 years followed by muscular rigidity and was diagnosed with parkinsonism at the age of 62 years when postural instability appeared. III‐10 had the same mutation pattern as I‐1, but there were no clinical manifestations of PD, probably due to her young age. The heterozygous deletion of exons 1 to 2 in the *PARK2* gene was detected in III‐2, III‐3, III‐5, and III‐11. All of them were <30 years of age and showed no similarities to the clinical features of their affected family members.

The pathological relevance of single heterozygous *PARK2* mutations has remained a hot topic of discussion, especially for late‐onset PD (Kay et al., 2007). In contrast to the data presented here, there is considerable evidence that a single *PARK2* mutation might be a risk factor for the late‐onset disease (Foroud et al., 2003). In addition, the clinical symptoms of the patients with single heterozygous *PARK2* mutations might be less severe than those with compound heterozygous mutations. Therefore, we need to follow‐up these individuals to investigate their clinical features.

In conclusion, we presented here novel compound heterozygous mutations in the *PARK2* gene, consisting of EX 1‐2 del and a splicing mutation c.619‐1 (G > C) in intron 6 and propose that the mutations were responsible for EOPD in a Chinese family, since other known PD‐related mutations could not be detected.

## CONFLICT OF INTEREST

None.

## AUTHOR CONTRIBUTIONS

Y.S, H.K, J.Z, and C.X are responsible for the conception and design of the study. Y.S, W.Z, and G.L were in charge of data collection and analysis of our study, besides, Y.S drafted the manuscript. Y.S and H.K were responsible for the critical revision of the manuscript. Y.S and C.X approved the final version of the manuscript.
