# Supplementary figures and images for: Novel compound heterozygous mutations in the PARK2 gene identified in a Chinese pedigree with early‐onset Parkinson's disease
Source: Brain Behav. 2017 Dec 19;8(1):e00901. doi: 10.1002/brb3.901 (PMC5853629; doi:10.1002/brb3.901)

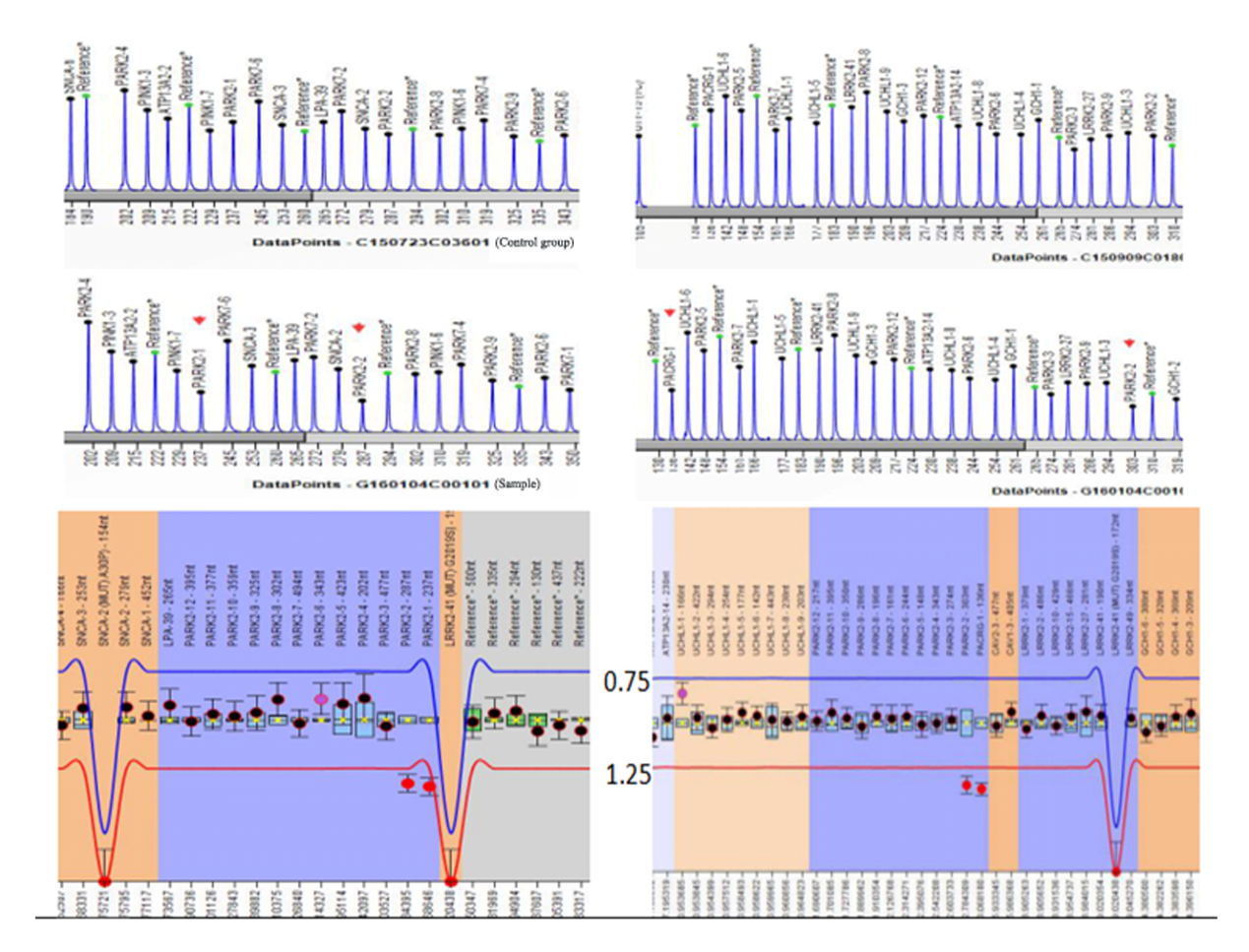

Supplement: Supplementary file 1 [file BRB3-8-e00901-s001.tif]
